# Association of Subjective Quality and Quantity of Sleep with Quality of Life among a General Population

**DOI:** 10.3390/ijerph182312835

**Published:** 2021-12-06

**Authors:** Kentaro Matsui, Takuya Yoshiike, Kentaro Nagao, Tomohiro Utsumi, Ayumi Tsuru, Rei Otsuki, Naoko Ayabe, Megumi Hazumi, Masahiro Suzuki, Kaori Saitoh, Sayaka Aritake-Okada, Yuichi Inoue, Kenichi Kuriyama

**Affiliations:** 1 Department of Laboratory Medicine, National Center Hospital, National Center of Neurology and Psychiatry, Tokyo 1878551, Japan; matsui.kentaro@ncnp.go.jp (K.M.); toyosawaa@ncnp.go.jp (A.T.); otsuki.rei@ncnp.go.jp (R.O.); 2 Department of Sleep-Wake Disorders, National Institute of Mental Health, National Center of Neurology and Psychiatry, Tokyo 1878553, Japan; yoshiike@ncnp.go.jp (T.Y.); knagao@ncnp.go.jp (K.N.); t-utsumi@ncnp.go.jp (T.U.); ayabe@ncnp.go.jp (N.A.); mhazumi@ncnp.go.jp (M.H.); 3 Japan Somnology Center, Neuropsychiatric Research Institute, Tokyo 1510053, Japan; inoue@somnology.com; 4 Department of Psychiatry, The Jikei University School of Medicine, Tokyo 1058461, Japan; 5 Department of Psychiatry, Nihon University School of Medicine, Tokyo 1738610, Japan; suzuki.masahiro94@nihon-u.ac.jp (M.S.); saitou.kaori79@nihon-u.ac.jp (K.S.); 6 Department of Regional Studies and Humanities, Faculty of Education and Human Studies, Akita University, Akita 0108502, Japan; 7 Department of Health Sciences, Saitama Prefectural University, Saitama 3438540, Japan; aritake-sayaka@spu.ac.jp; 8 Department of Somnology, Tokyo Medical University, Tokyo 1608402, Japan

**Keywords:** sleep quality, sleep duration, insomnia, quality of life

## Abstract

This study aimed to determine whether both subjective sleep quality and sleep duration are directly associated with quality of life (QOL), as well as indirectly associated with QOL through insomnia symptoms. Individuals aged 20–69 years without mental illness (*n* = 9305) were enrolled in this web-based cross-sectional survey. The Short Form-8 was used to assess physical and mental QOL. We used the Pittsburgh Sleep Quality Index (PSQI) and extracted items related to subjective sleep quality and sleep duration. Insomnia symptoms were also extracted from the PSQI. The hypothesized models were tested using structural equation modeling. Worse sleep quality, but not shorter sleep duration, was related to worse physical QOL. Both worse sleep quality and shorter sleep duration were related to worse mental QOL. Insomnia symptoms mediated these relationships. Subgroup analyses revealed a U-shaped relationship between sleep duration and physical/mental QOL. However, the relationship between sleep quality and physical/mental QOL was consistent regardless of sleep duration. The results suggest that subjective sleep quality has a more coherent association with QOL than subjective sleep duration. Because of its high feasibility, a questionnaire on overall sleep quality could be a useful indicator in future epidemiological studies of strategies for improving QOL.

## 1. Introduction

Quality of life (QOL), defined as “an individual’s perception of their position in life in the context of the culture in which they live and in relation to their goals, expectations, standards and concerns [1],” is a well-established concept in the fields of health and medicine. QOL is useful for predicting treatment and intervention success, and can be used as a qualitative health indicator that goes further than biomedical outcomes, such as curing disease and survival [2].

There is a close relationship between sleep and QOL in patients with a variety of physical and psychiatric illnesses and sleep disorders, and in the general population in all age groups [3]. Both quantitative and qualitative aspects of subjective sleep evaluation are reported to affect QOL. Several cross-sectional reports have indicated that short sleep duration is associated with lower QOL in the general population [4,5,6,7,8,9]. Simultaneously, a number of cross-sectional studies in the general population have reported that poor sleep quality is also associated with lower QOL [9,10,11,12,13,14,15,16]. In accordance with these findings, a recent longitudinal study reported that an increase in both sleep duration and sleep quality contributed to the improvement in QOL [17].

Although most epidemiological studies have assessed subjective sleep quality using the Pittsburgh Sleep Quality Index (PSQI) [18] as a standard measure [19], some studies have used insomnia symptoms as a measure of sleep quality [20,21]. The PSQI has also been used as a screening tool for insomnia [18,19]. However, insomnia symptoms are inherently different from sleep quality measures, given that sleep quality itself reflects a subjective feeling of restfulness and satisfaction with sleep [22]. Furthermore, studies that have assessed both subjective quality and duration of sleep, in which sleep quality was assessed using the PSQI, have shown that sleep quality is more strongly associated with health outcomes [9,17]; however, total PSQI scores include components related to both sleep quality and sleep duration [18], which might limit determining whether the qualitative or quantitative components of sleep are most important.

Questionnaires measuring overall sleep quality (C1, answer to the question “During the past month, how would you rate your sleep quality overall?”) and subjective sleep duration (C3, answer to the question “During the past month, how many hours of actual sleep did you get at night?”) were included in the PSQI as independent factors from other insomnia symptoms. Furthermore, because of its high feasibility and usability, a questionnaire on subjective sleep quality may be valuable for investigating the relationships between subjective quality of habitual sleep, QOL, and various health outcomes in epidemiological studies. We suspected that subjective quality and quantity of sleep might independently affect health-related outcomes because these reflect different aspects of an individual’s sleep experience; however, no studies have examined this using a single question on subjective sleep quality and sleep duration thus far. Therefore, we set out to conduct a preliminary cross-sectional investigation on how QOL is related to subjective sleep quality and quantity.

The purpose of the present study was to determine how subjective quality and duration of sleep influence physical and mental QOL in the general population without a history of psychiatric disorders. We hypothesized that both worse sleep quality and shorter sleep duration, respectively representing qualitative and quantitative assessments of sleep, would be directly associated with worse physical and mental QOL, as well as being indirectly associated with worse physical and mental QOL through insomnia symptoms. Our goal was to clarify which qualitative and quantitative sleep assessments were more closely associated with QOL. To address this question, we tested our hypothetical mediation model using structural equation modeling (SEM). Although a cross-sectional study design cannot express cause-effect relationships, SEM can reveal direct and indirect pathways of associations separately and express complicated relationships in a path diagram [23,24]. We performed the analysis not only for the entire sample but also for three subgroups categorized according to habitual sleep duration, based on the same hypothesis considering the possibility of a U-shaped relationship between sleep duration and QOL [5,6].

## 2. Materials and Methods

### 2.1. Participants

The present study was conducted as part of an epidemiological study to investigate excessive daytime sleepiness in subjects with suspected attention deficit hyperactivity disorder [8,25]. The ethics committee of the Neuropsychiatric Research Institute approved this study. The survey was conducted in February 2015 through Rakuten Research Inc., an online marketing research company with approximately 2.3 million registered Japanese users. The research firm randomly contacted selected individuals between the ages of 20 and 69 years old across the country, stratified by region, sex, and age, with an email including links to online questionnaires. It was clearly stated that this survey was related to sleep. Participants were provided with an informed consent form through the survey website. Among the 10,000 subjects who completed the questionnaires, 178 subjects whose data were missing and 517 subjects with self-reported ongoing treatment for mental illness were excluded. Finally, 9305 subjects were included in the analysis.

### 2.2. Study Measures

The questionnaire in this study included demographic information regarding age, sex, height, weight, occupation, smoking status, habitual alcohol ingestion, and the presence/absence of currently treated diseases [8,25]. Because body mass index was not normally distributed as a continuous variable, we divided it into five groups: less than 18.5 kg/m^2^, 18.5 to less than 25 kg/m^2^, 25 to less than 30 kg/m^2^, 30 to less than 35 kg/m^2^, and 35 kg/m^2^ or greater [26].

QOL was measured using the Japanese version of the Short Form-8 (SF-8) [27,28]. The SF-8 is an abbreviated version of an original 36-item health survey and contains psychometrically based physical and mental health summary measures. Physical QOL (physical component summary (PCS)) includes general health, physical function, role-physical, and bodily pain; mental QOL (mental component summary (MCS)) includes vitality, social function, mental health, and role-emotional. Scores of these domains were calculated according to standard methods [27].

The Japanese version of the Pittsburgh Sleep Quality Index (PSQI) [18,29] was applied to assess sleep quality, sleep duration, and insomnia symptoms. The PSQI is a standardized self-rated questionnaire developed to measure self-reported sleep disturbances. The 19 items are categorized into seven components and are evaluated with a score of 0 to 3. The PSQI components are as follows: subjective sleep quality (C1), sleep latency (C2), sleep duration (C3), habitual sleep efficiency (C4), sleep disturbances (C5), use of sleeping medication (C6), and daytime dysfunction (C7). The C5 score is calculated from answers to nine detailed questions, including a question regarding the frequency with which the respondents “wake up in the middle of the night or early morning” (C5a), which is also evaluated with a score of 0 to 3. We used the C1 score to evaluate sleep quality. C1 was scored according to the subjective rating of overall sleep quality during the past month as follows: very good (0), fairly good (1), fairly bad (2), and very bad (3). For the assessment of sleep duration, we used actual responses to the question on subjective sleep duration over the past month, which was reported in hours, rather than the C3 score itself. Given that difficulty falling asleep, difficulty maintaining sleep, and early-morning awakening are core symptoms of insomnia [30,31], we used the C2 score to assess difficulty initiating sleep and C5a score to assess difficulty maintaining sleep and/or waking up earlier than desired. C2 and C5a were scored according to the frequencies of difficulty in falling asleep within 30 min and waking up in the middle of the night or early morning, respectively, as follows: not during the last month (0), less than once a week (1), once or twice a week (2), and three or more times a week (3).

### 2.3. Statistical Analysis

**Figure 1:**
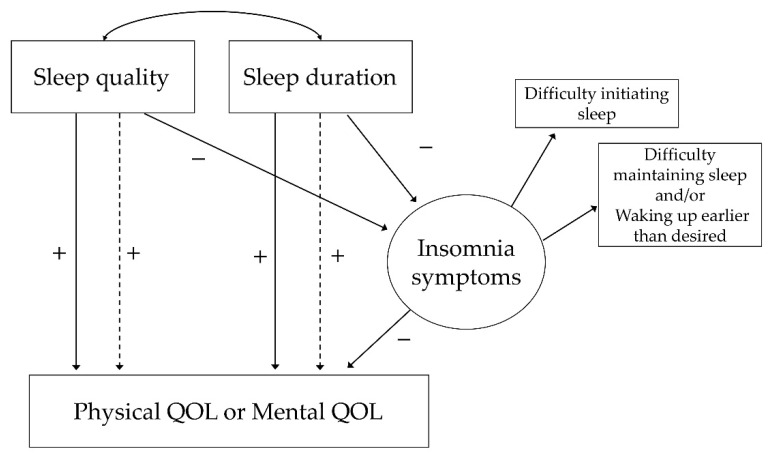
Hypothesized mediation model. Dashed lines are used to represent indirect effects, and solid lines are used to represent direct effects. QOL, quality of life.

Descriptive statistics and correlational analyses were performed using SPSS 26.0 J software (IBM Japan, Tokyo, Japan). Statistical significance was set at *p* < 0.001 to account for the large sample size and multiple comparisons. In our prior analysis, age, categorized BMI, and existence of currently treated diseases significantly correlated with PCS and age, sex, categorized BMI and existence of currently treated diseases significantly correlated with MCS (Spearman, *p* < 0.001 for all variables); therefore, we used these variables as covariates. The kurtosis of the individual variables was examined to confirm the normality of the distributions [32]. To examine the hypothesized relationships between sleep quality, sleep duration, insomnia symptoms, and physical or mental QOL, we performed a path analysis using SEM with Amos 26.0 (IBM Japan, Tokyo, Japan). The following hypotheses were proposed: (1) worse sleep quality and shorter sleep duration would be associated with worse physical/mental QOL and (2) insomnia symptoms would partially mediate the effect of sleep quality and sleep duration on physical/mental QOL. Two proposed path models were run separately for physical QOL and mental QOL to test our hypotheses (Figure 1). As subgroup analyses, these path analyses were also performed in each of the three groups: short sleep group (≤6 h, *n* = 2394), intermediate sleep group (>6 h to ≤8 h, *n* = 6105), and long sleep group (>8 h, *n* = 806) [33]. These models were determined as a good fit using the following criteria: chi-squared test of model fit (χ^2^), root mean square error of approximation (RMSEA < 0.08), non-normed fit index (NFI > 0.90), and comparative fit index (CFI > 0.90) [23,24]. A bootstrapping procedure using 5000 bootstrap samples was conducted to assess the statistical significance of the direct and indirect effects (mediation model) by calculating 95% confidence intervals (CIs), which were established by Preacher and Hayes [34]. We have reported the standardized coefficients. Thus, it was possible that paths that were constrained to be equal in the analysis of multiple groups had different beta coefficients based on the standardized metric.

## 3. Results

### 3.1. Correlation between Variables

Among the total sample (*n* = 9305), 4641 (49.9%) were women, 1832 (19.7%) were current smokers, 4541 (48.8%) had habitual alcohol consumption, 5192 (55.8%) had regular work, and 2738 (29.4%) were receiving treatment for physical disease. The means (standard deviation) of the variables assessed in the study were as follows: age, 45.8 (13.5) years; BMI, 22.2 (3.5) kg/m^2^; total PSQI score, 5.8 (3.5); PCS score, 48.6 (7.0); MCS score, 48.6 (7.6); C1 in the PSQI, 1.2 (0.7); C2 in the PSQI, 1.3 (1.6); C5a in the PSQI, 0.8 (1.1); and habitual sleep duration, 405.2 (63.1) min. Weak correlations were found between sleep quality and difficulty initiating sleep, difficulty maintaining sleep and/or waking up earlier than desired, physical QOL, and mental QOL. Correlations between sleep duration and difficulty initiating sleep or difficulty maintaining sleep and/or waking up earlier than desired were not significant. The correlations between sleep duration and physical QOL and mental QOL were significant, but both were considered to be negligible [35] (Table 1).

### 3.2. Mediation Analysis

**Figure 2:**
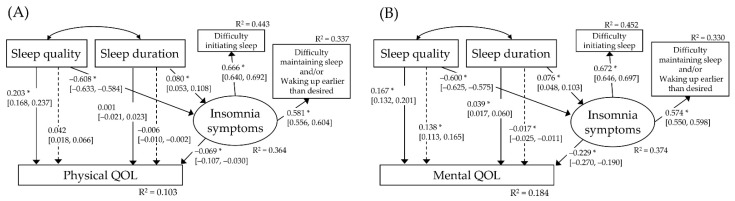
The hypothesized multiple mediator models examining the relationship of sleep quality and sleep duration to physical QOL (**A**) and mental QOL (**B**) for the entire sample (*n* = 9305). Standardized regression weights for each path are presented. Numbers in brackets represent 95% confidence intervals. Dashed lines are used to represent indirect effects, and solid lines are used to represent direct effects. The covariates (age, categorized body mass index, and existence of currently treated diseases for both PCS and MCS, and sex for MCS) were controlled in the equation, but they are not shown in the figure for the sake of brevity. * *p* < 0.001. QOL, quality of life. R^2^, R squared value.

We examined the hypotheses in two models to assess the influence of sleep quality and sleep duration on physical QOL (A) and mental QOL (B). All absolute kurtosis values were less than 3.00 [36]. The model-fit indices suggested that these models fitted the data well: (A) χ^2^ = 325.2, RMSEA = 0.058, CFI = 0.952, NFI = 0.951, and (B) χ^2^ = 357.4, RMSEA = 0.051, CFI = 0.957, NFI = 0.956. Specifically, the model accounted for 10.3% of the variance in physical QOL (A) and 18.4% of the variance in mental QOL (B). The SEM results are shown in Figure 2.

In model (A), path coefficients suggested that worse sleep quality was directly associated with worse physical QOL (β = 0.203, 95% CI 0.168 to 0.237; *p* < 0.001). The results of bootstrapping indicated a trend for an indirect association between worse sleep quality and worse physical QOL (β = 0.042, 95% CI 0.018 to 0.066; *p* = 0.001). Meanwhile, sleep duration was not significantly associated with physical QOL. Moreover, the results of the bootstrapping indicated a trend for an indirect association between longer sleep duration and worse physical QOL (β = −0.006, 95% CI −0.010 to −0.002; *p* = 0.001). In model (B), path coefficients suggested that worse sleep quality was directly associated with worse mental QOL (β = 0.167, 95% CI 0.132 to 0.201; *p* < 0.001). The results of bootstrapping indicated an indirect association between worse sleep quality and worse mental QOL (β = 0.136, 95% CI 0.113 to 0.165; *p* < 0.001). Shorter sleep duration was also directly associated with worse mental QOL (β = 0.039, 95% CI 0.017 to 0.060; *p* < 0.001). However, the results of bootstrapping indicated an indirect association between longer sleep duration and worse mental QOL (β = −0.017, 95% CI −0.025 to −0.011; *p* < 0.001), which showed the opposite direction from the direct association.

**Figure 3:**
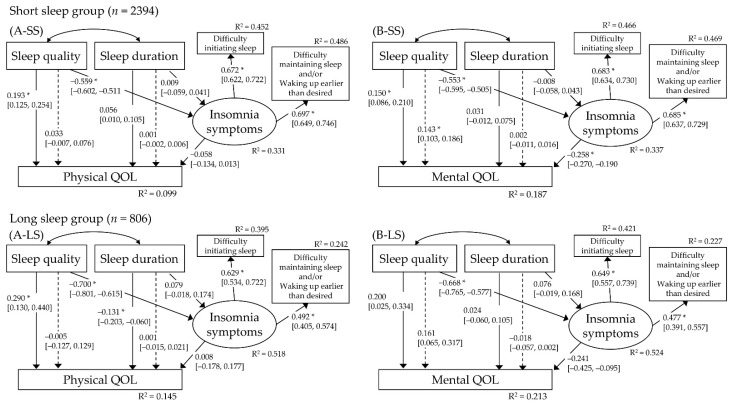
The hypothesized multiple mediator models examining the relationship of sleep quality and sleep duration to physical QOL and mental QOL for the short sleep group (A-SS and B-SS, respectively, *n* = 2394) and long sleep group (A-LS and B-LS, respectively, *n* = 806). The analyses performed were the same as those shown in Figure 2. * *p* < 0.001. QOL, quality of life. R^2^, R squared value.

Demographic and clinical data for the three subgroups (short sleep group, intermediate sleep group, and long sleep group) are shown in Table S1. All path analyses performed in each group showed a good model fit (Table S2). The relationship between sleep quality and physical/mental QOL was mostly consistent among the three subgroups. In the short sleep group, unlike the results in the total sample, (1) there was a trend for a direct correlation between shorter sleep duration and worse physical QOL (β = 0.056, 95% CI 0.010 to 0.105; *p* = 0.005), (2) the correlation between sleep duration and insomnia symptoms was negligible in both models (A-SS, *p* = 0.689 and B-SS, *p* = 0.745), and (3) the correlation between insomnia symptoms and physical QOL was not significant (*p* = 0.067) (Figure 3). In the long sleep group, unlike the results in the total sample, (1) there was a significant direct correlation between longer sleep duration and worse physical QOL (β = −0.131, 95% CI −0.203 to −0.060; *p* < 0.001), (2) the correlation between sleep duration and insomnia symptoms was not significant in either model (A-LS, *p* = 0.069 and B-LS, *p* = 0.077), and (3) the correlation between insomnia symptoms and physical QOL was negligible (*p* = 0.927) (Figure 3). The results of the path analysis in the intermediate sleep group were identical to those for the entire sample (Table S2).

## 4. Discussion

This is the first study to examine the relationships between subjective sleep quality/quantity and QOL in the general population using PSQI sub-items. The present study confirmed the hypothesis that overall sleep quality as a subjective measure, which is one of the items of the PSQI, significantly correlated with both physical and mental QOL, as well as being indirectly related through insomnia symptoms. The relationships between sleep quality and physical or mental QOL seemed more apparent than the relationships between sleep duration and physical or mental QOL, given that sleep quality had a consistent association with both physical and mental QOL across the subgroups of sleep duration. Some studies have assessed sleep quality using the C1 score of the PSQI as in this study [37,38,39], but none of them have examined the contrast between quality and duration of sleep. Because this is a preliminarily study suggesting an association between QOL in a cross-sectional design, future studies should clarify how subjective sleep quality and quantity affect future health-related indicators.

Contrary to our hypothesis, there was a clear association between longer sleep duration and insomnia symptoms; moreover, longer sleep duration was indirectly associated with poorer mental QOL via insomnia symptoms. Those with long subjective sleep duration in this study can be described as those who spent a long time in bed; for them, sleep restriction may alleviate insomnia symptoms [40]. Sleep restriction therapy is part of a package of cognitive behavioral therapy for insomnia (CBT-I) [41,42], but it has been suggested to be effective against insomnia symptoms when used alone [43]. Given the contribution of CBT-I to the improvement of QOL [44,45], the possible contribution of shortened sleep duration to improved mental and physical QOL in the long sleep group can be interpreted as being consistent with these previous reports.

Among the entire sample, shorter sleep duration was directly associated with worse mental QOL. However, contrary to our hypothesis, sleep duration was not directly associated with physical QOL. This result is consistent with some previous literature [46,47], but differs from reports that both physical and mental QOL are associated with sleep duration [4,7]. Notably, the results of the subgroup analyses suggested that in the short sleep group, longer sleep duration tended to correlate with better physical QOL, while in the long sleep group, longer sleep duration significantly correlated with worse physical QOL. These findings suggest that the relationship between sleep duration and physical QOL was not linear but U-shaped, which is consistent with some previous reports [5,6]. However, regardless of the length of sleep duration, the relationship between sleep duration and mental QOL was weak. These differences between physical and mental QOL in their relationship to sleep duration are similar to those found in studies of the general population in the United Kingdom and United States [4]. Although the findings of this study suggest that the relationship between sleep duration and mental QOL is modest, considering the well-documented importance of sleep duration with respect to lower QOL [4,5,6,7,8,9,46], the health risks of habitual short sleep duration should still be emphasized.

The current study had several limitations. First, this study used a cross-sectional research design in which causality could not be established. Future research should consider using an experimental or longitudinal research design to examine the associations between sleep quality, sleep duration and QOL. Second, difficulty initiating sleep, difficulty maintaining sleep, and early morning awakening are three independent items in the diagnostic criteria for insomnia [30,31]; however, in the current study, difficulty maintaining sleep and early morning awakening were evaluated as a single item. This limitation was due to the design of this study, which examined the hypothesis using subscales of the PSQI. Third, this study used an internet-based survey. It has been suggested that internet users may have more sleep problems and sleep less, potentially biasing the sample [48]. In addition, because the potential participants were informed in advance that the survey was about sleep, it is possible that people interested in sleep actively chose to participate in this study. Fourth, we excluded subjects who were receiving treatment for mental illness, which was based on self-report and not a direct diagnosis. We may have overlooked participants with mental illness when it was undiagnosed or undeclared.

## 5. Conclusions

The results of the present study suggested that subjective sleep quality was significantly associated with both physical and mental QOL and that sleep duration was significantly associated with mental QOL. Because sleep quality was associated with both physical and mental QOL regardless of sleep duration, it may have the potential to influence subsequent health-related outcomes. Future longitudinal studies should examine the association between sleep quality/quantity and health outcomes using objective indicators.

## Figures and Tables

**Table 1:** Bivariate correlations (*r*) of descriptive statistics (*n* = 9305).

	1	2	3	4	5	6	7	8	9	10	11	12	13
1. Age	-												
2. Sex	0.003	-											
3. Categorized BMI	0.127 *	−0.269 *	-										
4. Current smoker	0.014	−0.194 *	0.056 *	-									
5. Habitual alcohol consumption	0.073 *	−0.238 *	0.054 *	0.158 *	-								
6. Regular worker	−0.195 *	−0.442 *	0.115 *	0.158 *	0.192 *	-							
7. Existence of currently treated diseases	0.308 *	−0.023	0.121 *	−0.023	0.012	−0.067 *	-						
8. Physical QOL (PCS)	−0.054 *	0.000	−0.065 *	−0.032	0.029	0.001	−0.190 *	-					
9. Mental QOL (MCS)	0.242 *	−0.046 *	0.066 *	−0.013	0.016	−0.029	0.045 *	−0.045 *	-				
10. Difficulty initiating sleep (C2 in PSQI)	−0.098 *	0.101 *	−0.015	0.040 *	−0.042 *	−0.096 *	0.025	−0.132 *	−0.229 *	-			
11. Difficulty maintaining sleep and/or waking up earlier than desired (C5a in PSQI)	0.107 *	0.047 *	−0.008	−0.014	0.043 *	−0.056 *	0.098 *	−0.140 *	−0.166 *	0.378 *	-		
12. Sleep quality (C1 in PSQI)	−0.103 *	0.058 *	−0.005	0.034	−0.017	−0.008	0.022	−0.254 *	−0.323 *	0.374 *	0.322 *	-	
13. Habitual sleep duration (min)	−0.019	0.028	−0.044 *	−0.044 *	−0.003	−0.090 *	0.010	0.058 *	0.078 *	−0.008	−0.008	−0.209 *	-

Sex, 0 = female, 1 = male. Categorized BMI, 1 = less than 18.5 kg/m^2^, 2 = 18.5 to less than 25 kg/m^2^, 3 = 25 to less than 30 kg/m^2^, 4 = 30 to less than 35 kg/m^2^, 5 = 35 kg/m^2^ or greater. BMI, body mass index; QOL, quality of life; PCS, physical component summary of the SF-8; MCS, mental component summary of the SF-8; PSQI, Pittsburgh Sleep Quality Index. * *p* < 0.001.

## Data Availability

The data will be shared on reasonable request to the corresponding author.

## Supplementary Materials

The following are available online at https://www.mdpi.com/article/10.3390/ijerph182312835/s1, Table S1: Demographic and clinical data in the short, intermediate, and long sleep groups, Table S2: Model fit indices and standardized direct and indirect relations of variables in the entire sample and subgroups.
