# Supplementary material for: Association of Subjective Quality and Quantity of Sleep with Quality of Life among a General Population
Source: Int J Environ Res Public Health. 2021 Dec 6;18(23):12835. doi: 10.3390/ijerph182312835 (PMC8657737; doi:10.3390/ijerph182312835)
Supplement: Supplementary file 1 [file ijerph-18-12835-s001.zip › ijerph-1459107-supplementary.pdf]

Supplementary Material

Table S1. Demographic and clinical data between short, intermediate, and long sleep groups.

	Short sleep group (N = 1,782)	Intermediate sleep group (N = 6,172)	Long sleep group (N = 1,351)
Age, median (range), year	45 (20–69)	45 (20–69)	45 (20–69)
Female, N (%)	883 (49.6)	3,042 (49.3)	716 (53.0)
BMI, median (range), kg/m^2	22.0 (10.2–50.4)	21.6 (11.2–61)	21.5 (15.6–52.1)
Current smoker, N (%)	407 (22.8)	1,209 (19.6)	216 (16.0)
Habitual alcohol ingestion, N (%)	880 (49.4)	3,041 (49.3)	620 (45.9)
Regular worker, N (%)	1,107 (62.1)	3,514 (56.9)	571 (42.3)
Existence of currently treated diseases, N (%)	511 (28.7)	1,789 (29.0)	438 (32.4)
Physical QOL (PCS), median (range), point	49.5 (14.1–67.5)	50.2 (11.2–65.7)	50.3 (10.8–70.6)
Mental QOL (MCS), median (range), point	48.8 (13.1–65.2)	50.3 (15.5–68.3)	51.4 (10.6–70)
Difficulty initiating sleep (C2 in PSQI), median (range), point	1 (0–6)	1 (0–6)	1 (0–6)
Difficulty maintaining sleep and/or waking up earlier than desired (C5a in PSQI), median (range), point	0 (0–3)	0 (0–3)	0 (0–3)
Sleep quality (C1 in PSQI), median (range), point	1 (0–3)	1 (0–3)	1 (0–3)
Habitual sleep duration, median (range), min	325.7 (180.0–355.7)	411.4 (360.0–475.7)	488.6 (480–917.1)

BMI, body mass index; QOL, quality of life; PCS, physical component summary of the SF-8; MCS, mental component summary of the SF-8; PSQI, Pittsburgh Sleep Quality Index.

Table S2. Model fit indices and standardized direct and indirect relations of variables between the entire sample and subgroups.

Mediation model on physical QOL (A)

	Total (N = 9,305)	Short sleep group (N = 1,782)	Intermediate sleep group (N = 6,172)	Long sleep group (N = 1,351)
Model Fit				
χ2	434.2	115.4	300.1	99.5
RMSEA	0.048	0.041	0.046	0.047
CFI	0.955	0.950	0.951	0.950
NFI	0.953	0.936	0.948	0.935
Path analysis				
Sleep Quality –> Physical QOL				
Direct association	0.203***	0.165***	0.194***	0.267***
Indirect association	0.041**	0.039	0.044**	−0.002
Sleep Duration –> Physical QOL				
Direct association	−0.002	0.078***	0.018	−0.099***
Indirect association	−0.005**	0.002	−0.004**	0.000
Insomnia symptoms –> Physical QOL	−0.068***	−0.075*	−0.073**	0.003
Sleep quality –> Insomnia symptoms	−0.598***	−0.527***	−0.601***	−0.631***
Sleep duration –> Insomnia symptoms	0.077***	−0.029	0.053***	0.096***
Difficulty initiating sleep –> Insomnia symptoms	0.677***	0.688***	0.658***	0.644***
Difficulty maintaining sleep and/or waking up earlier than desired –> Insomnia symptoms	0.571***	0.691***	0.551***	0.526***

Mediation model on Mental QOL (B)

	Total (N = 9,305)	Short sleep group (N = 1,782)	Intermediate sleep group (N = 6,172)	Long sleep group (N = 1,351)
Model Fit				
χ2	434.2	111.8	293.5	99.4
RMSEA	0.046	0.039	0.045	0.046
CFI	0.958	0.957	0.955	0.954
NFI	0.956	0.943	0.952	0.940
Path analysis				
Sleep Quality –> Mental QOL				
Direct association	0.169***	0.132***	0.171***	0.188***
Indirect association	0.136***	0.156***	0.154***	0.144***
Sleep Duration –> Mental QOL				
Direct association	0.040***	0.040	0.030*	0.027
Indirect association	−0.017***	0.009	−0.011**	−0.022**
Insomnia symptoms –> Mental QOL	−0.227***	−0.296***	−0.202***	−0.229***
Sleep quality –> Insomnia symptoms	−0.598***	−0.526***	−0.600***	−0.630***
Sleep duration –> Insomnia symptoms	0.077***	−0.029	0.053***	0.096***
Difficulty initiating sleep –> Insomnia symptoms	0.675***	0.683***	0.659***	0.647***
Difficulty maintaining sleep and/or waking up earlier than desired –> Insomnia symptoms	0.572***	0.693***	0.550***	0.522***

RMSEA, root mean square error of approximation; NFI, non-normed fit index; CFI, comparative fit index; QOL, quality of life. *$p < 0.05$, **$p < 0.01$, ***$p < 0.001$.
